# The prevalence of Coxiella burnetii in ticks and animals in Slovenia

**DOI:** 10.1186/s12917-019-2130-3

**Published:** 2019-10-25

**Authors:** Nataša Knap, Diana Žele, Urška Glinšek Biškup, Tatjana Avšič-Županc, Gorazd Vengušt

**Affiliations:** 1 Faculty of Medicine, Institute of Microbiology and Immunology, University of Ljubljana, Zaloska 4, 1000 Ljubljana, Slovenia; 2 Veterinary Faculty, Institute for Pathology, Wild Animals, Fishes and Bees, University of Ljubljana, Gerbiceva 60, 1000 Ljubljana, Slovenia

**Keywords:** *Coxiella burnetii*, Slovenia, Ticks, Domestic and wild ungulates, Real-time PCR, Seroprevalence

## Abstract

**Background:**

The obligate intracellular bacterium *Coxiella burnetii* causes globally distributed zoonotic Q fever. Ruminant livestock are common reservoirs of *C. burnetii*. *Coxiella burnetii* are shed in large numbers in the waste of infected animals and are transmitted by inhalation of contaminated aerosols. This study was conducted to evaluate the prevalence of *C. burnetii* infection in domestic animals and ticks in areas of Slovenia associated with a history of Q fever outbreaks.

**Results:**

A total of 701 ticks were collected and identified from vegetation, domestic animals and wild animals. *C. burnetii* DNA was detected in 17 out of 701 (2.4%) ticks. No *C. burnetii* DNA was found in male ticks. Ticks that tested positive in the PCR-based assay were most commonly sampled from wild deer (5.09%), followed by ticks collected from domestic animals (1.16%) and ticks collected by flagging vegetation (0.79%). Additionally, 150 animal blood samples were investigated for the presence of *C. burnetii-*specific antibodies and pathogen DNA. The presence of pathogen DNA was confirmed in 14 out of 150 (9.3%) blood samples, while specific antibodies were detected in sera from 60 out of 150 (40.4%) animals.

**Conclusions:**

Our results indicate that ticks, although not the primary source of the bacteria, are infected with *C. burnetii* and may represent a potential source of infection for humans and animals. Ticks collected from animals were most likely found to harbor *C. burnetii* DNA, and the infection was not lost during molting. The persistence and distribution of pathogens in cattle and sheep indicates that *C. burnetii* is constantly present in Slovenia.

## Background

*C. burnetii* is an obligate intracellular organism phylogenetically related to Gammaproteobacteria and is the causative agent of Q fever, a globally distributed zoonosis. *C. burnetii* infections have been reported throughout the world in livestock, other domestic and wild mammals, birds and a wide variety of ticks [1].

*Although* ticks are not considered essential in the natural cycle of *C. burnetii* in livestock, they form part of the transmission cycle of the organism in wildlife [1–3]. The microorganism multiplies in the gut cells of ticks, and large numbers of *C. burnetii* are shed in tick feces [4]. Maurin and Raoult (1999) reported over 40 tick species to be naturally infected with *C. burnetii*, including genera of *Ixodes, Haemaphysalis*, *Rhipicephalus* and *Dermacentor* ticks [5].

The primary reservoirs of *C*. *burnetii* are sheep, goats and cattle [6, 7]. Animals that are often naturally infected usually do not show typical symptoms except during pregnancy, when abortions and other reproductive disorders could occur. Thus, diagnosis of Q fever based on clinical symptoms or postmortem examination is very difficult or almost impossible due to unspecific or missing symptoms or lesions caused by this disease [8]. The microorganism is shed in high numbers into the environment from amniotic fluids and placenta during parturition. Infected animals excrete C. *burnetii* in the milk, urine, and feces [9–11]. Although infection in animals is generally considered subclinical, it has been associated with abortion, stillbirth or infertility, reproductive disorders and mastitis [1, 12–14]. In humans, Q fever is a highly variable disease, ranging from asymptomatic infection to fatal chronic infective endocarditis. The most commonly identified sources of human infection are farm animals such as cattle, goats, and sheep.

The role of wildlife, namely, wild and farmed deer, in the transmission of this pathogen has not been thoroughly investigated. Although evidence of *C. burnetii* infection has been confirmed in wild and farmed deer, there are no reports to date linking exposure to deer species with human Q fever cases [15, 16]. Generally, infection follows the inhalation of contaminated aerosol particles derived from heavily infected placentas or rarely through the processing of the consumption of raw animal products [1, 7].

In comparison to other rickettsial species, *C. burnetii* withstands environmental conditions, chemicals and dehydration. Because of its stability in the environment, close contact with the herd is not required for infection [1, 17]. Reducing exposure to the microorganism is difficult because animals with no detectable *C. burnetii* specific antibodies can shed the bacteria at parturition [1]. The scarcity of studies and clinically unapparent infection might be reasons for the limited information regarding the prevalence of *C. burnetii* in domestic and wild animals, as well as the rate of infection of ticks. To determine the risk of infection, the sources and routes of transmission must be identified. To our knowledge, *C. burnetii* infection, including risk factors, such as exposure to farm and wild animals, and ticks, has not yet been characterized in Slovenia.

The objective of the present study was to estimate the prevalence of *C. burnetii* infection using serological and PCR analyses of domestic animals and in questing and fed ticks in the territory of Slovenia.

## Results

Seven hundred and one tick samples, of which 626 *Ixodes ricinus*, 65 *Haemaphysalis punctata* and 10 *Dermacentor reticulatus* were identified, collected by flagging vegetation and from farm animals, were tested for the presence of the pathogen. *C. burnetii* DNA was detected in 16 *I. ricinus* samples and 1 *H. punctata* sample. Four of the positive *I. ricinus* samples were nymphs or adult female ticks collected from the vegetation (Table 1). Five tick samples in which *C. burnetii* DNA was detected were collected from farm animals (4 *I. ricinus* and 1 *H.punctata*) and 8 samples from *I. ricinus* ticks collected from wildlife. The difference between the number of positive ticks collected from animals and from vegetation was statistically significant (*p* = 4.75*10–7), with significantly more positive ticks sampled from animals than on vegetation. The overall prevalence of *C. burnetii* infection in questing ticks in Slovenia was calculated as 0.8% (2.6% in female adults and 0.65% in nymphs). Nevertheless, when adjusted according to fed state (questing vs. fed), no significant difference was confirmed in the rate of infection between tick stages (*p* = 0.546832). No pathogen DNA was detected in adult male tick samples. Questing ticks with confirmed *C. burnetii* DNA were sampled at 4 out of 8 selected locations. Ticks with confirmed infection were sampled from animals (all sheep) from 3 out of 4 locations (Table 2). No significant differences were confirmed between locations (*p* > 0.05). In addition to the ticks sampled from domestic animals, we also investigated ticks sampled from wild deer. Eight out of 157 sampled *I. ricinus* ticks carried *C. burnetii* DNA (Table 1). No significant difference was confirmed between the infection rate of domestic animals originating fed ticks and ticks sampled from wild animals (χ^2^-test, *p* = 0.258995).

**Table 1:** Detection of *C. burnetii* DNA in ticks according to tick species, stage and sex and origin of ticks

Tick species and stage	*Ixodes ricinus*				*Haemaphysalis punctata*			*Dermacentor reticulatus*	Total
Origin of tick	Larvae	Nymphs	Adult (Female)	Adult (Male)	Nymphs	Adult (Female)	Adult (Male)	Adult (Female)	
Vegetation	0/21 (0%)	0/265 (0%)	2/70 (2.8%)	2/86 (2.3%)	0/42 (0%)	0/8 (0%)	0/9 (0%)	–	4/501 (2.8%)
Cattle	–	1/1 (100%)	3/34 (8.8%)	0/2 (0%)	–	1/3 (33%)	0/3 (0%)	–	5/43 (11.6%)
Wildlife	–	2/15 (13.3%)	6/115 (5.2%)	0/17 (0%)	–	–	–	0/10 (0%)	8/157 (5.1%)
Total	0/21 (0%)	3/281 (1.1%)	11/219 (5.0%)	2/105 (1.9%)	0/42 (0%)	1/11 (9.0%)	0/12 (0%)	0/10 (0%)	17/701 (2.4%)

**Table 2:** Detection of *C. burnetii* DNA in tick (questing and fed) and animal samples and antibody detection in animal sera

Region	Goriška		Notranjsko-kraška	Pomurska	Podravska	Obalno-kraška		Gorenjska	Total
Location	Čiginj	Volče	Dolenja vas	Mačkovci	Maribor	Senožeče	Vremščica	Žirovnica	
The number of ticks pools positive for *C. burnetii*/ total number of ticks sampled from vegetation	1/65 (1.52%)	0/44 (0%)	0/149 (0%)	0/93 (0%)	0/1 (0%)	1/17 (5.10%)	1/80 (1.24%)	1/52 (1.90%)	4/501 (0.79%)
The number of ticks with positive for *C. burnetii*/ total number of ticks sampled from animals	–	3/29 (10.3%)	1/4 (25%)	–	–	0/6 (0%)	1/4 (25%)	–	5/43 (11.6%)
Adjacent farm animals	sheep	sheep	sheep	cattle	cattle	sheep	sheep	cattle	
The number of animal blood samples with detected *C. burnetii*/ total number of animal blood samples	5/20 (25%)	1/20 (5%)	0/20 (0%)	2/21 (9.5%)	0/10 (0%)	2/20 (10%)	2/20 (10%)	2/19 (10.5%)	14/150 (9.3%)
The number of animal blood samples with detected antibodies against *C. burnetii*/ total number of animal blood samples	4/20 (20%)	4/20 (20%)	12/20 (60%)	18/21 (85.7%)	5/10 (50%)	5/20 (25%)	12/20 (60%)	1/19 (5.2%)	61/150 (40.4%)
The number of ticks positive for *C. burnetii*/ total number of ticks sampled from deer	0/24 (0%)		2/10 (20%)	0/33 (0%)	2/33 (6%)	4/35 (11.4%)		0/22 (0%)	8/157 (5.09%)

Molecular analysis of animal blood samples showed the presence of the pathogen DNA in 6 out of 8 locations while antibodies against *C. burnetii* were found in all of the selected locations (Table 2). The presence of *C. burnetii* was found in sheep and cattle in all sampled locations in Slovenia. The overall seroprevalence of *C. burnetii* was 36% (36/100) in sheep and 46% (23/50) in cattle. However, the difference between sheep and cows in the prevalence rate was not statistically significant (*p* = 0.406821). When comparing the prevalence of infection between locations, we confirmed that significantly more animals came into contact with *C. burnetii* at location 3 (Mačkovci, χ^2^-test, *p* = 0.031721), and significantly fewer animals were infected by the pathogen at location 8 (Žirovnica, χ^2^-test, *p* = 0.017544) compared with other locations.

## Discussion

During recent years, Q fever outbreaks in Europe have indicated a very pressing need to study the disease and its causative agent, *C. burnetii*. Up to five Q fever cases confirmed annually in Slovenia indicate a long-term presence of these bacteria in Slovenia. Despite the occurrence of sporadic cases, little is known about the incidence of the bacterium and its geographic distribution in domestic and wild animals. Therefore, a broader approach has been used to determine the presence of the bacterium in Slovenian cattle and sheep farms, both in animal samples and in samples from ticks.

The wide distribution of antibodies against *C. burnetii* in cattle and sheep indicates that this pathogen is endemic throughout Slovenia (Table 2). At least one animal on every farm harbored antibodies against the investigated pathogen, and almost 90% of animals on some farms have been in contact with *C. burnetii.* In comparison, the seroprevalence in domestic animals was similar to that reported from neighboring Italy and was slightly lower than that reported from Slovakia [21, 22]. The seroprevalence in sheep reported here is similar to that in neighboring Italy [22] and slightly lower [21] than that in Slovakia, while the seroprevalence in cattle in this study was slightly lower than that from neighboring Hungary [23] and considerably lower than that from Denmark [24].

Studies detecting antibody carriers against *C. burnetii* demonstrate previous exposure to the pathogen, not current shedding of the pathogen; nevertheless, they are useful for epidemiological determination of endemic areas [5]. Shedding of *C. burnetii* has been demonstrated by the detection of bacterial DNA from placental tissue, feces, vaginal fluid and milk of infected animals [25]. Sheep, goats and cattle shed organisms into the environment at high concentrations, where they can survive for months [5]. Human infection is due to inhalation of aerosolized bacteria (from the birth process) or consumption of raw milk.

The relatively high seroprevalence reported in this study was expected considering that farms with a previous history of abortion were chosen for the study. In the investigated regions no significant outbreaks have been recognized in proximity to sheep and cattle farms during the time of our study, but our results indicate that the Q fever causative agent is actively present in these areas, and special precautions should be considered for people working on or close to the investigated areas. In fact, one sampling site was the source of a significant outbreak in 2007, where 33 veterinary students and two teachers contracted Q fever during a training course on a sheep farm and outbreak control measures were implicated [26].

Major aspects of human Q fever outbreaks are connected to domestic and wild ruminants, but they may also contribute to the maintenance of *C. burnetii* in nature. Particularly due to the increase of deer farming worldwide, it is important to understand whether wildlife represents a serious risk for human and animal health. However, there is still no direct evidence connecting exposure to deer species with human Q fever cases [15]. But it has been suggested that exposure of hunters during game carcass dressing in the field may represent potential zoonotic risk [16]. The role of wildlife in the epidemiology of *C. burnetii* is largely unknown; therefore, the impact of particular control measures is rather uncertain.

Ticks in which *C. burnetii* DNA was detected were collected in 4 out of 8 locations, where the presence of the bacterium has been established by serological screening of infected animals. The role of ticks in the *C. burnetii* transmission cycle has been discussed in the past, and the bacterium has been confirmed in ticks in a number of countries in Europe [27–31]. However, it is evident that the ticks are not primary source of infection of domestic animals and humans, which are infected by inhalation of contaminated aerosols or dust containing *C. burnetii* shed by infected animals [32]. Nevertheless, the ticks are infected with the bacteria and may represent another potential source of bacterial transmission, particularly for wild animal populations and domestic animals that have spent substantial time in pastures, where large numbers of ticks can be found. Additionally, the presence of *C. burnetii* DNA in two tick species, *I. ricinus* and *H. punctata*, is in accordance with previous studies, which have found that the pathogen is present in more than 40 tick species and is not limited to a single species [29] . Significantly more ticks collected from animals were found to harbor *C. burnetii* DNA compared to questing ticks, indicating bacterial transfer during feeding or enhanced bacterial proliferation after the onset of feeding. The number of unfed ticks that carried the bacterial DNA indicates that the infection is likely not lost during moulting. There was no significant difference between bacterial detection in questing adult ticks and nymphs indicating the possibility of transtadial transmission. Neverthless although circumstantial evidence indicates the possible role of ticks in *C. burnetii* transmission and circulation in nature, additional studies are needed to confirm the vector capacity of *I. ricinus* and *H. punctata* to transmit *C. burnetii.* We used IS1111 for the detection of *C. burnetii* since it was originally only described in this species. In recent years, it has become evident that a wide range of IS1111 analogs exist in other bacteria, such as in *Coxiella-*like endosymbionts of ticks. This raises the question of the specificity of the diagnostic test used for field studies of Q fever epidemiology; a lack of specificity may lead to an overestimation of *C. burnetii* presence [33]. Unfortunately, the number of bacterial DNA copies detected in ticks was too low to enable successful confirmation by sequencing of *rpoB* and 16S rRNA.

This study suggests the permanent circulation of *C. burnetii* in nature. The constant presence of this pathologic agent among domestic animals and ticks is of concern. Further studies on the presence of *C. burnetii* among wildlife are needed to elucidate the role of wildlife in the epidemiology of Q fever.

## Conclusions

The wide distribution of antibodies against *C. burnetii* in cattle and sheep indicates that this pathogen is endemic throughout the studied region. The obtained results indicate that the Q fever causative agent is actively present in these areas, in ticks, and cattle and sheep. Therefore, special precautions should be considered for people working in or close to the investigated areas to minimize the risk of infection.

## Methods

### Tick and animal blood sampling

**Fig. 1:**
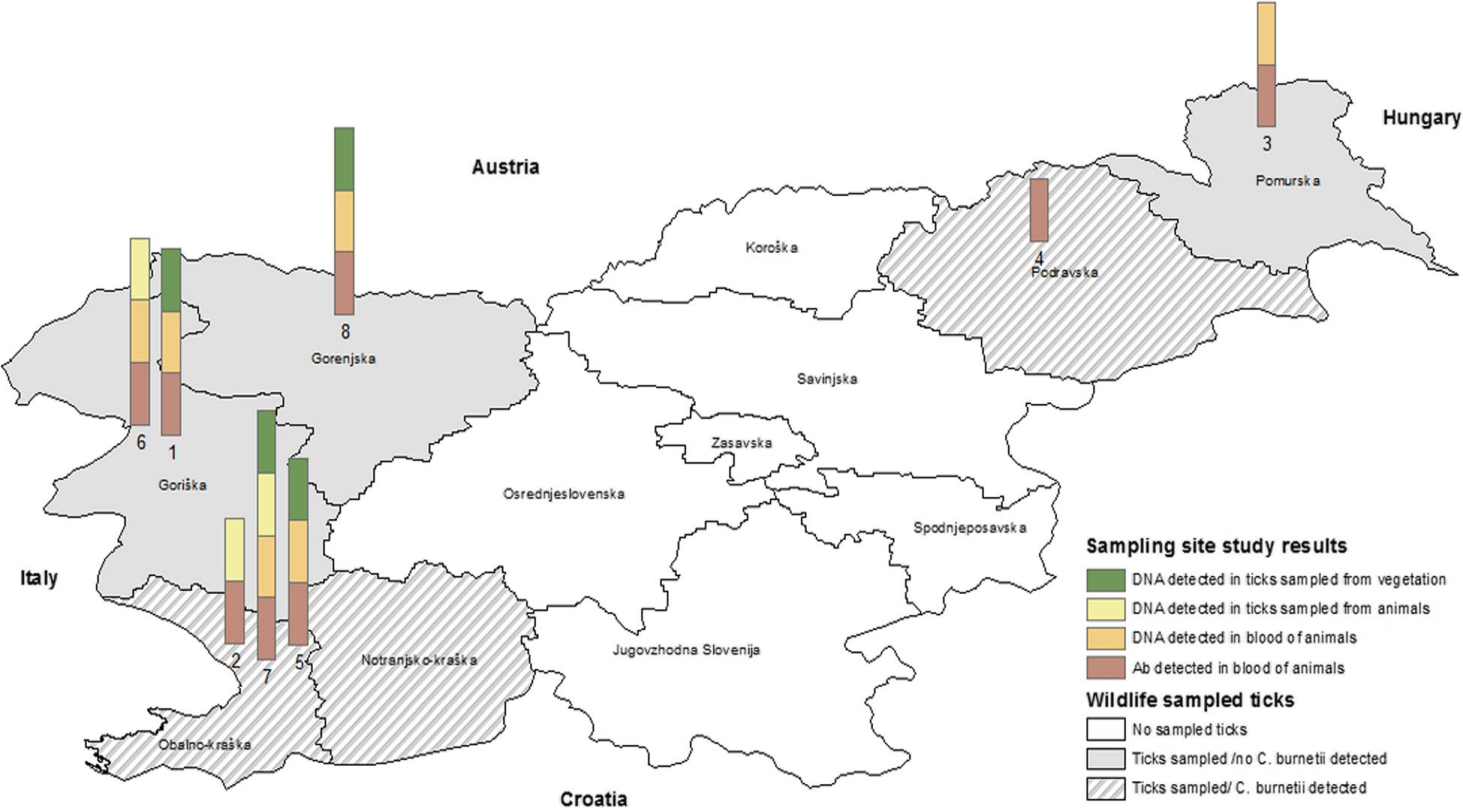
Data on tick and cattle infection incidence/prevalence on sampling locations (1–8) and data on regional presence of infected ticks sampled from free living deer. (Source: Figure was created by the authors with ArcGIS 10.4). * Sampling sites: 1 – Čiginj, 2 – Dolenja vas, 3 – Mačkovci, 4 – Maribor, 5 – Senožeče, 6 – Volče, 7 – Vremščica, 8 – Žirovnica

Ticks were sampled at eight locations in Slovenia in the spring and autumn of 2009. The climate in Slovenia is continental. The winters are cold and the summers are warm, but at the coastal areas, there is submediterranean climate. More than half of Slovenia’s land surface is covered with forest, other mainly natural areas, natural grassland, wetlands, water bodies and open spaces with little or no vegetation [18]. Ticks were collected by flagging the lower vegetation with a 1 m^2^ white cotton cloth at peripheral areas of forests and pastureland. Sampling locations were selected in areas where previous infections of farm animals with *C. burnetii* had been confirmed (Table 2, Fig. 1). In addition to flagging vegetation, ticks were collected from domestic animals on adjacent farms. Considering that red deer (*Cervus elaphus*) is one of the most common species of wild ruminants in Slovenia, ticks were additionally sampled from carcasses of wild red deer in six Slovenian regions (Table 2, Fig. 1). The species, stage and sex of the collected ticks were determined morphologically [19], and the ticks were decontaminated in 70% ethanol and sterile double-distilled water. Samples of questing ticks were pooled into groups of 10 nymphs or 5 adults according to tick species and sex. Ticks collected from animals were stored and analyzed individually. Ticks were stored at − 20 °C until further analysis. Furthermore, blood samples were taken from a section of domestic animals (cows, sheep) on the selected farms. Blood samples (*n* = 150) were collected in May and in October from adult cattle (*n* = 50) or sheep (*n* = 100) on 8 farms from herds with a history of abortions and reproductive disorders (Fig. 1). Blood samples from the caudal vein (cattle) or jugular vein (sheep) were collected into 10 ml sterile serum separation tubes (Vacuette; Greiner Bio-One, Kremsmunster, Austria) and transported to the laboratory where sera were obtained, and further serological examinations were performed.

### Pathogen detection

Tick samples (90 pools of 501 individuals sampled from vegetation and 43 and 157 individula ticks sampled from farm animals and wild animals respectively) and blood samples from farm animals were used for DNA extraction. Tick samples were homogenized using Tissue Lyser (Retsch for Qiagen, Hilden, Germany). DNA from all sets of samples was extracted according to the manufacturer’s instructions with the BioSprint 15 DNA Blood Kit (Qiagen, Hilden, Germany).

DNA samples were screened for *C. burnetii* DNA by probe-specific real-time PCR detecting a 66 bp portion of the transposase gene (IS1111) following the protocol published by Panning et al. [20].

Additionally, a serological survey of animal plasma samples was performed using the commercial CHEKIT Q-Fever Antibody ELISA Test Kit (IDEXX, Liebefeld-Bern, Switzerland). The samples were tested for antibodies against *C. burnetii* based on inactivated phase I and phase II antigens. The optical density (OD) of the samples was averaged and corrected by subtracting the OD of the negative control. The obtained results were expressed as S/*P* values and estimated as the ratio between OD of the sample (S) and the OD of the positive control (P) included in the test kit. S/*P* values equal to or greater than 40% were considered positive, and S/P values below 40% were considered negative, according to the manufacturers instructions.

### Statistical analysis

The software package SPSS Statistics IBM 19 (© IBM Corporation, Somers, New York, USA) was used for statistical analysis. PooledInfRate software version 3.0 (Microsoft® Excel Add-In developed by Brad Biggerstaff; CDC, Fort Collins, CO) was used to establish infection rates from pooled sample data. The prevalence of *C. burnetii* was analyzed according to the following variables: tick sampling site, stage and species using chi-square test or Fisher’s exact test. P values of < 0.05 were considered statistically significant.

## Data Availability

Data supporting the conclusions of this article are included in the article. Raw data for calculation of method validation, tables, and figures are available from the corresponding author upon request.

## Abbreviations

*C. burnetii*: *Coxiella burnetii*
*D. reticulatus*: *Dermacentor reticulatus*
*H. punctata*: *Haemaphysalis punctata*
*I. ricinus*: *Ixodes ricinus*
OD: optical density
P: positive control
S: sample
